# Quantifying the Performance of P-Type Transparent Conducting Oxides by Experimental Methods

**DOI:** 10.3390/ma10091019

**Published:** 2017-09-01

**Authors:** Karsten Fleischer, Emma Norton, Daragh Mullarkey, David Caffrey, Igor V. Shvets

**Affiliations:** School of Physics, Trinity College, The University of Dublin, D02PD91 Dublin 2, Ireland; nortonem@tcd.ie (E.N.); mullarkd@tcd.ie (D.M.); dcaffrey@tcd.ie (D.C.); ivchvets@tcd.ie (I.V.S.)

**Keywords:** transparent conducting oxide, TCO, p-type, figure of merit, material screening, TFT, solar cell, selective contact

## Abstract

Screening for potential new materials with experimental and theoretical methods has led to the discovery of many promising candidate materials for p-type transparent conducting oxides. It is difficult to reliably assess a good p-type transparent conducting oxide (TCO) from limited information available at an early experimental stage. In this paper we discuss the influence of sample thickness on simple transmission measurements and how the sample thickness can skew the commonly used figure of merit of TCOs and their estimated band gap. We discuss this using copper-deficient CuCrO${}_{2}$ as an example, as it was already shown to be a good p-type TCO grown at low temperatures. We outline a modified figure of merit reducing thickness-dependent errors, as well as how modern ab initio screening methods can be used to augment experimental methods to assess new materials for potential applications as p-type TCOs, p-channel transparent thin film transistors, and selective contacts in solar cells.

## 1. Introduction

There are a wide range of transparent conducting oxides (TCOs) available for today’s optoelectronic applications [1,2,3,4,5,6,7]. Typical commercially employed TCOs are highly n-type doped wide bandgap oxide materials such as indium tin oxide (In${}_{2}$O${}_{3}$:Sn, ITO), doped zinc oxide (ZnO:Al, ZnO:Ga, ZnO:In), fluorinated tin oxide (SnO${}_{2}$:F, FTO), and amorphous indium gallium zinc oxide (InGaZnO${}_{4}$, IGZO). Despite their commercial success, extensive research is being undertaken to find new candidate materials in particular, to replace In-rich TCOs and to find viable hole conducting counterparts (p-type TCOs). The former is driven by increasing indium costs and the desire for high-mobility TCOs to minimise free carrier absorption for solar cell applications, while the latter is required for novel fully-transparent optoelectronics. p-type TCOs have been reported since 1997 starting with CuAlO${}_{2}$ by Kawazoe et al. [8]. Since these initial findings, many other oxides showing p-type conductivity have been found, ranging from other oxides in the delafossite crystal structure family [8,9,10,11] to spinels [12,13,14], perovskites [15], and corundum-type oxides [16,17,18].

While many of these p-type oxides have been used in laboratory demonstration devices, their performance in terms of transparency, conductivity, and hole mobility is still severely lacking, and experimental and computational screening for new and better-performing p-type TCOs continues. For both experimental as well as computational screening methods, it is crucial to identify key material parameters reliably—sometimes with limited experimental information—in order to assess a given material’s potential as a p-type transparent oxide. For computational screening, the established procedure is to calculate the band structure of a material, and to assess its bandgap and valence band dispersion to estimate the hole mobility [14,19,20,21,22]. In a second more computationally expensive step, native defect formation energies are calculated to assess if the material can be successfully doped p-type or if native point defects can lead to acceptor compensation [14,20,23]. This high degree of compensation is the case for all of the known commercial n-type TCOs, where acceptor defects—typically oxygen vacancies—prevent sufficient p-type doping [24,25,26].

When experimentally screening for best properties, it is preferable to derive a first measure of quality from a compact set of key properties, allowing for a quick assessment without the need for extensive characterisation. Such a measure of quality is often referred to as a figure of merit (FOM). In the context of TCOs, such a figure of merit has been established to relate the overall absorption within the material to its conductivity [27]. In this paper, we will review its use in the literature and highlight potential shortcomings of this approach when the figure of merit is used incorrectly.

## 2. An Assessment of the Commonly Used Figure of Merit

The key parameters for assessing a transparent conducting oxide are its conductivity σ and absorption coefficient α. These two properties can be combined by defining a figure of merit $F_{TR}$ as [27,28]:

(1) $$F_{TR}=\frac{\sigma }{\alpha }=-\frac{1}{R_{s}\ln\left(T+R\right)}$$

In this form, a theoretically thickness-independent measure of quality (with units of Siemens) can be evaluated solely by measuring a thin film’s sheet resistance ($R_{s}$) as well as its transmittance (*T*) and reflectance (*R*). In many ways, this is an ideal quantity for experimental screening, as it can be derived from relatively simple measurements, without requiring any detailed knowledge of, for example, the film thickness. In perfect epitaxial systems where the microstructure of the TCO is not affected by the sample thickness, $F_{TR}$ is a reliable quantity to be used in comparing the performance of p-type TCOs. In polycrystalline samples grown on glass films, for example (as is common for screening methods), the use of $F_{TR}$ favours thicker films, as the crystalline quality typically improves with layer thickness. Consequently, *T* as well as $R_{s}$ improve for thicker layers of an otherwise identical material. This issue can be easily addressed by comparing films with similar thickness, by quantifying the change in $F_{TR}$ for different film thickness, or as a bare minimum always explicitly stating the film thickness for which results are given.

A second problem arises as reflectance measurements are less frequently employed for material screening. Simple ultraviolet-visible (UV-VIS) spectrophotometers are typically used in many such environments which lack the capability to perform reflectance measurements. If the figure of merit is used in the simplified form of [28,29]:

(2) $$F_{T}=-\frac{1}{R_{s}\ln\left(T\right)}$$

it loses its thickness independence, making reliable comparison between samples with differing thicknesses difficult. Furthermore, using only transmission data can be problematic, as it is difficult to distinguish between unwanted absorption losses and acceptable reflection losses. This will be further discussed in Section 2.1.

A third issue is that the figure of merit alone does not refer to a materials band gap or hole mobility, which are of crucial importance for optoelectronic devices. As a result a high figure of merit will not always translate to a enhanced efficacy of a particular device. The details of these issues and how to assess them will be discussed in Section 2.2.

### 2.1. Comparing Known p-Type TCO Materials—The Issue of Limited Information

**Figure 1:**
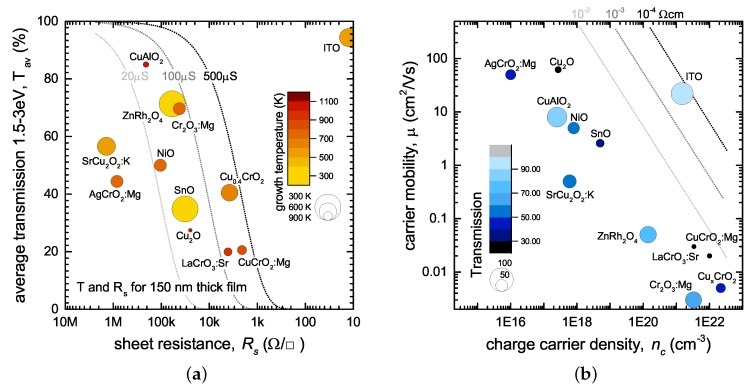
(**a**) Comparison of the literature data of average transmission and sheet resistance of a range of p-type transparent conducting oxides (TCOs). The reported literature data have been adjusted to account for varying thickness between samples, and the average transmission was taken over an energy range of 1.5–3 eV. Dotted lines indicate “constant figure of merit” lines. The size of the points scales inversely with growth temperature (or post-annealing temperature if required), as excessive temperature also hampers the ability of materials to be successfully used in applications; (**b**) The reported carrier concentration and hole mobility for the same set of samples, with the size of the point proportional to the transparency. Lines of equal resistivity are shown, illustrating the poor performance of today’s p-type TCOs compared to ITO (Data sources are listed in Table 1).

One key problem of comparing different new p-type TCOs discovered recently is that the figure of merit is often used without specifying if $F_{TR}$ or $F_{T}$ have been used, and more importantly, in many cases peak transmission values are used rather than average transmission values over the spectral range of interest for displays and solar cells (1.5–3 eV). Therefore, many low band gap materials have been reported with a high FOM, despite possessing limited transparency in the visible range. In Figure 1a, a number of common p-type TCOs have been re-assessed by plotting their sheet resistance vs. their transparency, following the approach of Zhang et al. [15]. In addition, we have added secondary information such as the growth temperature required, and in Figure 1b plotted carrier concentration vs. mobility in the same films. We have only included materials where the full transmission spectra were available (to calculate the average transmission), and where the electrical measurements have been performed on the same films as optical measurements.

Comparing different p-type TCOs in such a way already reveals that two materials with the same figure of merit can have substantially different properties, as illustrated by the dotted lines in Figure 1, which show calculated lines of “constant FOM”. Films with 20% transmission can have a similar FOM to those with 80%, providing their conductivity is high enough. Depending on the specific application and device geometry, such low transparency might well be unacceptable, rendering the material unsuitable despite a high figure of merit. Indeed, it was already commented on in one of the original works on defining the FOM that the comparison should ultimately be undertaken with a specific device structure (and also layer thickness) in mind [28].

In crystalline material, depending on the growth method and conditions, grain sizes and therefore electrical properties can differ significantly between thinner and μm-thick films. In this case, deriving a *representative* FOM for the material can be problematic, and it is harder to distinguish between systematic problems of the FOM calculation and real material changes. To address the former, we will discuss the thickness dependence of the TCOs’ figure of merit by examining data from recently developed nanocrystalline copper-deficient Cu${}_{0.4}$CrO${}_{2}$ [30,31,32,33]. In the case of Cu${}_{0.4}$CrO${}_{2}$, the large copper deficiency results in a poor crystallinity of the material, and consequently the material’s microstructure and electrical properties are not altered by growing thicker layers. Hence, the FOM can be easily assessed for different sample thickness by calculating transmission and reflection data, as well as sheet resistance from the material’s optical constants (n,k) and measured conductivity (σ). The latter data have been measured for a well-characterised sample grown by spray pyrolysis, where the sample thickness was measured accurately by X-ray reflection (XRR).

**Figure 2:**
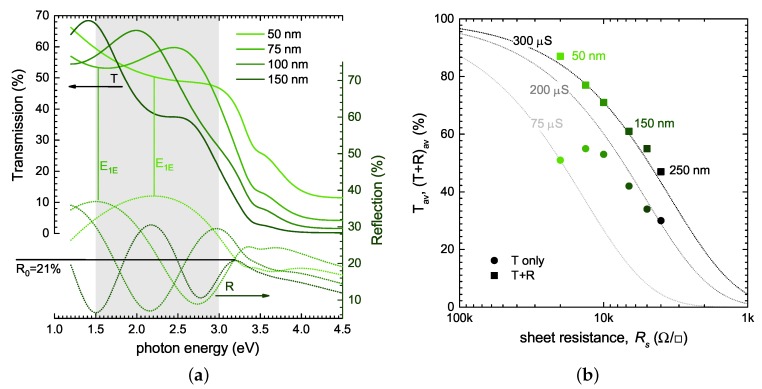
(**a**) Modelled transmission (—) and reflection (···) data of Cu${}_{0.5}$CrO${}_{2}$ thin films on glass. The grey spectral region was used to calculate the average transmission and reflection; (**b**) The position of each film on the $T_{av}$ vs. $R_{s}$ graph using only transmission data (●) and *T* + *R* data (*■*).

Figure 2 shows the modelled transmission and reflectance spectra for Cu${}_{0.4}$CrO${}_{2}$ thin films on glass for a thickness range of 50 to 150 nm, as well as the position of Cu${}_{0.4}$CrO${}_{2}$ films in the *T* vs. $R_{s}$ graph for thicknesses up to 250 nm. These optical models clearly show that the reflectance can be as high as 30% for very thin films once the refractive index of the material is substantially higher than that of glass. The root cause of the thickness dependence is the Fabry–Pérot interference fringes in the transparent region of the TCO. For the discussed case of Cu${}_{0.4}$CrO${}_{2}$, it can be seen in Figure 2a that the reflection from a 50 nm thin film is significantly higher than those of higher thickness, as the broad maxima of the interference fringe coincides with the evaluated spectral range. Consequently, the average transmission in the desired region $T_{av}$ was found to be much lower than for a 100 nm film where the reflectance is dominated by a minima of the interference fringe. For film thicknesses of greater than 150 nm, several interference fringes were found in the spectral region of interest and the evaluated average reflectance is therefore more consistent, approaching the expected reflectance of the material in bulk form. For Cu${}_{0.4}$CrO${}_{2}$ this is about 21% using the average refractive index of *n* = 2.7 and the Fresnel equation to calculate the reflectance of a bulk material at normal incidence ($R_{0}$):

(3) $$R_{0}={\left(\frac{\left(n-1\right)}{\left(n+1\right)}\right)}^{2}$$

Many candidate p-type TCOs have a much higher refractive index *n* than typical n-type TCOs. In the visible region, *n* of Cu${}_{0.4}$CrO${}_{2}$ is 2.5–2.8—much larger than, the one of ZnO (∼1.9–2), for example. Consequently, from Equation (3), it can be seen that the measured transmission of such thin films will not exceed 70% as a result of the air/TCO interface—even if there is no parasitic absorption within the thin film.

Using these modelled transmission and reflection data, one can now also illustrate the error introduced into the simplified figure of merit ($F_{T}$) once only transmission data are considered. For the thickness range investigated, $F_{T}$ for Cu${}_{0.4}$CrO${}_{2}$ ranges from just 75 μS to 200 μS, as illustrated in Figure 2b. Once reflectance data are included, $F_{TR}$ varies significantly less between measurements on films with different thickness, and is found to be ≈300 μS. We have to stress that the reflectance losses are not necessarily detrimental, as in a real device they will be mitigated once the p-type TCO is embedded between, for example, metal or n-type TCO contacts and absorber layers. In these cases, reflectance losses will be different than in the case of a p-type TCO/air interface measured during typical screening methods, and can even have a beneficial effect as an internal anti-reflective layer [34].

### 2.2. Measuring a Robust Figure of Merit in Screening Methods

So far, we have outlined that using only transmission data to calculate $F_{T}$ rather than $F_{TR}$ can lead to significant errors. Fortunately, even in cases where reflection cannot be practically measured due to instrumental limitations, it is possible to at least estimate the full thickness-independent figure of merit $F_{TR}$ by adding $R_{0}$ (as calculated by Equation (3)) to the measured average transmission. However, for new materials, the refractive index *n* would also be unknown. *n*, however, can be suitably derived for the purpose of correction of $F_{T}$ using the following methods:

**Direct measurement:** Ideally, the thin film can be analysed by ellipsometry to measure the complex refractive index n+ik. In that case, the absorption coefficient α=4πk/λ is known and the conductivity σ can also be calculated from the sheet resistance $R_{s}$ and the sample thickness derived from the ellipsometric model. Hence, $F_{TR}$ can already be fully derived by (1) without any additional approximations.

**Estimation by analysing interference fringes:** In general, transmission datasets do not contain enough information to derive the refractive index without additional information. However, for screening methods of transparent materials it is possible to at least estimate the refractive index by analysing the Fabry–Pérot interference fringes. The method analyses the position of the interference maxima, minima, as well as their envelope in measured transmission data, and can be employed if a homogeneous, smooth, thin film is considered [35,36]. This method requires more than one interference fringe to be present, and therefore needs not only a sufficient spectral range of the instrument, but also a thicker sample, with—crucially—very low absorption. Using Cu${}_{0.4}$CrO${}_{2}$ data from Figure 2, it can be seen that for a typical p-type TCO such as Cu${}_{0.4}$CrO${}_{2}$ , a sample thickness of more than 150 nm is required to clearly observe more than one interference extrema. At the same time, the absorbance in this thickness range already significantly dampens the second transmission maximum. If the sample thickness *d* is known from other methods, the energetic position of the first extrema (maximum in reflectance, or minima in transmission) , $E_{1E}$ (see Figure 2a) can be used to estimate *n* using the following equation, provided $n_{TCO}>n_{substrate}$:

(4) $$n_{TCO}=\frac{\lambda_{1E}}{4d}=\frac{ch}{E_{1E}4d}\approx \frac{1239.8}{E_{1E}4d}$$

We have to stress that such an estimation should be performed by using at least two films with different sample thickness to avoid the interpretation of absorption features (e.g., due to defects or weak dipole-forbidden transitions) as interference minima. As illustrated, the interference minima in transmission data are much less pronounced than in reflectance data, and the determination of their position from transmission data alone is only possible for film thickness well above 50 nm. For thicker layers (d>100 nm), the position of two adjacent extrema (if *T* is plotted versus photon energy) can be used, as the spacing between extrema is directly proportional to *n* [35]. We must highlight that this analysis should only be used to estimate the refractive index for correcting the figure of merit. It should not be used to extract full optical constants of p-type TCOs, as the core assumption of $n^{2}\gg k^{2}$ is typically not valid in many of these materials. Hence, the extracted spectral form and absolute values will have larger systematic errors than in the cases the methods were originally developed for. The error of this method is also directly linked to the sample thickness; if the latter is not determined independently, *n* will be over or underestimated likewise.

**Estimation using ab initio optical constants or material density:** With the enormous progress in recent years regarding the ab initio description of material properties and the wide availability of these data in public databases [37,38,39], it becomes feasible to use such ab initio data to augment experimental data if direct measurements are unavailable. In selected cases, the dielectric function of a material can already be well reproduced; hence, the thin film reflection can be calculated or approximated using (3).

Computational screening for p-type TCOs often uses variants of density functional theory (DFT), which include the electron–hole self interaction in terms of an expansion of the single particle Green’s function *G* and the Coulomb interaction *W* (GW-correction) [40]. The calculated material band gaps are therefore significantly more accurate than in standard local density approximation (DFT-LDA) or generalised gradient approximation (DFT-GGA). Even if self energy corrections are used, the calculated dielectric functions—while providing good qualitative descriptions—are still lacking accuracy to quantitatively describe the entire spectral response of a thin film. However, in the relevant transparent region, the dielectric response is featureless below the band gap, and hence the ab initio data are sufficient to estimate *n* and consequently the reflectance at normal incidence $R_{0}$. In the example of our nanocrystalline Cu${}_{0.4}$CrO${}_{2}$ , no direct DFT calculation of the highly defective structure has been performed. However GW-corrected calculations of closely related crystalline delafossite CuCrO${}_{2}$, or spinel Cr${}_{2}$CuO${}_{4}$ are found in such databases, predicting a static *n* of 2.49 and 2.51 [39], respectively, in good agreement with the experimental value of 2.7. The small discrepancy arises due to the relatively small bandgap of the material (≈2.5 eV), which will add already to the refractive index around 2 eV due to non-negligible dispersion.

Even in the case that self-energy corrected DFT is not available the DFT-LDA (or DFT-GGA) data can be used in a first-order approximation, even if the refractive index (or static dielectric constant) has not been calculated in any form and band gaps are severely underestimated—the atomic positions in these calculations are nevertheless very accurate. Consequently, the material density can be easily calculated from the DFT data. For oxides, as discussed here, the empirical Gladstone-Dale relationship [51] and more modern modifications of it [52,53] link the refractive index of a mineral to its density $\rho_{d}$ and can be used to estimate $n=K\rho {}_{d}+1$. The Gladstone–Dale constant *K* has been tabulated for many minerals, and largely depends on the cations and their coordination [52].

The use of any of the DFT-based methods to evaluate *n* is also useful to evaluate potential errors in the sample thickness, as it should be consistent with *n* derived from the evaluation of the interference fringes. Large discrepancies could indicate deviations in the sample thickness.

To summarise this section, even if experimental data during material screening lacks the crucial reflectance data to assess the performance of the p-type TCO with the figure of merit $F_{TR}$, it should at least be approximated by estimating the normal incidence reflectance using the methods outlined above. Table 1 lists the materials shown in Figure 1, including $F_{TR}$ if the published information were sufficient to calculate the latter. In all cases, we also give values for $F_{T}$ and how the inclusion of an estimated normal incidence reflectance leads to a more representative and thickness-independent $F_{TR_{0}}$.

## 3. Application-Specific Considerations

The figure of merit in itself is a useful tool to quickly assess promising candidate p-type TCOs. However, the reduction to the mere ratio of absorption and resistivity does not reflect the specific needs for different applications—an issue common to all transparent conducting materials [6]. As seen in Figure 1, materials with a similar figure of merit can show significant differences (e.g., in the actual transmission). In cases where a high transmission is paramount (e.g., illuminated transparent contacts in solar cells), a thick layer of CuCrO${}_{2}$:Mg, for example, would be detrimental, despite the highest $F_{TR}$ of all p-type TCOs. Other applications (e.g., p-channel thin film transistors TFTs) require specific carrier concentrations and high hole mobilities—information not directly accessible by the figure of merit.

**Figure 3:**
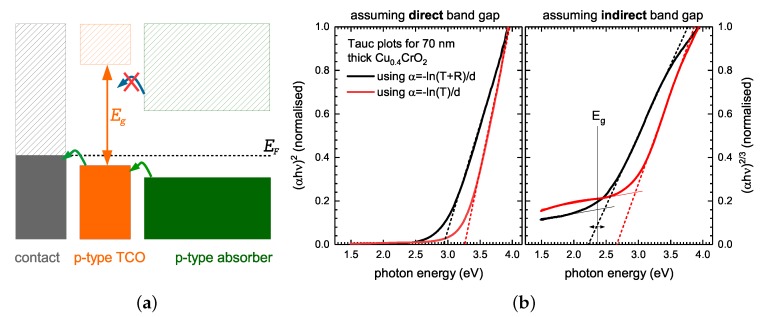
(**a**) Schematic illustration of the preferred band alignment for a hole transport/electron blocking layer; (**b**) Tauc plots for a 70 nm-thick Cu${}_{0.4}$CrO${}_{2}$ thin film using both transmission and reflection data (black lines) as well as transmission data only (red lines). The Tauc plot for both an assumed direct and an indirect band gap are shown. Only by ab initio information predicting an indirect band gap can the actual band gap of the material be correctly identified.

**Selective contacts:** One main application of p-type TCOs are so-called hole transport or electron blocking layers in thin film solar cells or organic light emitting diodes (OLEDs). Their purpose is to minimise shunting of the diode by electrons from the active region reaching the anode contact metal. The purpose of such a layer is to maximise hole extraction (or injection in case of OLEDs) by having a valence band structure well aligned with the high work function contact and the valence band of the active absorber (emitter) material. At the same time, it should create a significant transport barrier for electrons from the absorber conduction band to reduce device shunting. Figure 3a illustrates the requirements schematically.

The typical layer thickness for such selective contacts ranges from 10–30 nm. Transport in these layers is governed by the valence band position of the p-type TCO and its offset to the absorber layer valence band. The sheet resistance of the p-type TCO in itself is of secondary importance—particularly for very thin layers. More crucial in this case is a high transmittance to minimise parasitic absorption within the contact and a large band gap ($E_{g}$) to maximise the conduction band offset and hence barrier height in the conduction band. Hence, for solar cell application the $E_{g}$ of the p-type TCO should be well above 2 eV and even higher for OLED applications, as the band gap of the active region is already in the 2–2.5 eV range.

Experimentally, the band gap is typically determined by Tauc plots to evaluate the absorption coefficient as determined from the transmission and reflectance measurement [54]. However, evaluation with the Tauc method *requires prior knowledge of the type of gap* to use the correct exponent *r* in the Tauc equation ${\left(\alpha h\nu \right)}^{1/r}$. Figure 3b illustrates the evaluation for a 70 nm thick Cu${}_{0.4}$CrO${}_{2}$ film, assuming either a direct (r=1/2) or indirect (r=3/2) band gap. Analysing these data could either be interpreted as a 2.95 ± 0.05 eV direct or 2.4 ± 0.1 eV indirect gap. GW-corrected DFT calculations for crystalline CuCrO${}_{2}$ and Cr${}_{2}$CuO${}_{4}$ predict indirect band gaps of 2.5 eV and 1.4 eV, respectively [39]. It is therefore reasonable to also assume an indirect band gap for the copper-deficient material, with broad tail states due to the defective nature of the material. This example clearly illustrates that using the most common Tauc evaluation with r=1/2 would severely overestimate the band gap in the case of Cu${}_{0.4}$CrO${}_{2}$.

As with the figure of merit, additional complications arise if no reflectance data are available. Figure 3b also shows the Tauc plots for α calculated from transmission data alone. It can be seen that the resulting linear fits to evaluate the band gap differ significantly from the correct evaluation using T+R data. The issue unfortunately arises prominently for thin smooth films of a weakly absorbing material, which are usually the *desired properties* for TCOs. In this case, Fabry–Pérot interference structures are superimposed to the absorption edge. In Figure 2a it can be seen that for a 75 nm-thick film, the maximum in transmission due to interference is in the range of 2.5–3 eV—the spectral range where the Tauc-fit is performed. For new unknown materials and in cases where only transmission data are available, it is therefore crucial to compare films with different thickness to quantify the magnitude of this issue. Without appropriate reflectance data, it is also not feasible to a priori interpret differences in the fitted intercept in the Tauc plot for different film thickness as a change in band gap. Even if reflectance data are available, it is worth noting that the Tauc method will only correctly give band gap values in the case of parabolic valence and conduction bands, resulting in an absorption feature of a three-dimensional bulk critical point [55]. In the case of flat bands, 1D-bulk critical points, and for strong excitonic absorption, the Tauc plot will always underestimate band gaps. For specific systems (e.g., ZnO and a-Si), the specific problems of Tauc plots have been previously discussed [56,57]. For p-type TCOs (in particular within screening experiments for new materials), these systematic issues relating to Tauc plots have to be considered and need to be minimised by the experimental design by either always including reflectance data, or at least systematically studying any thickness dependence and comparing results using different Tauc exponents with ab-initio calculations.

**p-channel transparent thin film transistors:** A second potential application for p-type TCOs are fully-transparent thin film transistors (TTFT). Individual transistors made of n-type TCOs are already commercially used in driving circuits for OLED displays. The availability of both n- and p-channel transistors is desirable for complementary logic circuits. Hence, considerable research focus is dedicated to p-channel TTFTs. For transistor applications, the figure of merit is again helpful to identify suitable candidate materials, as high transparency and good conductivity are desired characteristics. However, crucial parameters for TFT device performance are not considered by $F_{TR}$. Namely, hole mobilities (μ) well above 1 cm${}^{2}$/Vs are essential to be competitive with hydrogenated amorphous silicon-based electronics. Secondly, the carrier concentration ($n_{c}$) needs to be easily controllable, and a highly conductive material should have hole concentrations on the order of $10^{18}$–$10^{19}$ cm${}^{-3}$.

Hence, knowledge of the carrier concentration and mobility for new materials found in experimental screening methods is essential to assess the full potential of a new p-type TCO. Carrier mobilities are typically measured by Hall measurements in either van der Pauw or Hall bar geometry. Due to the much more resistive nature of even the best performing p-type TCO thin films typically investigated in material screening, great care has to be taken that measured Hall voltages and hence mobilities are not affected by systematic errors of Hall measurements themselves. The Hall mobility should be measured for different bias currents and magnetic fields as standard practice for highly resistive samples [58]. Many p-type TCOs—and chromium-based TCOs in particular [15,18,42,59,60]—have limited mobilities due to a polaronic nature and consequently have a hopping-type conduction mechanism. In such cases, direct current (DC)-Hall measurements will not give consistent information, and other methods of measuring carrier mobilities must be applied. For CuCrO${}_{2}$, both AC-Hall measurements as well as estimates from thermopower measurements and assuming variable range or small polaron hopping have been used. Estimations assuming band conduction, and fits of resistance versus temperature measurements to link the measured carrier activation energy to the carrier concentration should be avoided. As previously shown in the case of a related p-type TCO (Cr${}_{2}$O${}_{3}$:Mg), such an estimation gives a mobility of 0.4 cm${}^{2}$/Vs, while Hall measurements were not able to confirm this [18]. Analysing the same dataset, assuming small polaron hopping, the carrier activation energy is then related to the energy barrier for the hopping process, not to the carrier generation. For new materials with limited information on the type of transport, it is therefore prudent to only publish directly measured Hall mobilities, following rigorous measuring procedures required for high resistive samples [58], high field measurements [44], or AC-Hall data.

## 4. Materials and Methods

**Sample synthesis:** All p-type TCOs (Cu${}_{0.4}$CrO${}_{2}$) directly measured for this study were synthesised by spray pyrolysis using solutions of 0.025 M chromium acetyl-acetonate and 0.0075 M copper acetyl-acetonate in methanol. The precursor solution was sprayed with an air blast nozzle (PNR MAD 825), liquid flow of 1.7 mL/min in a nitrogen/compressed air mixture with 5% total oxygen content at 15 L/min. The substrate (glass microslides) were kept at 350 ${}^{\circ }$C during the deposition. The growth rate under these conditions was found to be 9 nm/min.

The Cr${}_{2-x}$Mg${}_{x}$O${}_{3}$ deposited via magnetron sputtering for this study was synthesised via two methodologies. The first was co-deposition from a metallic Cr and ceramic MgO target in a reactive O${}_{2}$ atmosphere. This deposition was performed at 750 ${}^{\circ }$C and 0.45 Pa at 9% O${}_{2}$ content, and films had a 13% Mg content with 120 W applied to the Cr and 115 W applied to the MgO. The second was the use of a single Cr${}_{2-x}$Mg${}_{x}$O${}_{3}$ target with 8% Mg content. Deposited films had 8% Mg content, and were deposited with 150 W power at 720 ${}^{\circ }$C and 0.45 Pa in pure Ar atmosphere.

**XRD and thickness determination:** All investigated Cu${}_{0.4}$CrO${}_{2}$ and Cr${}_{2-x}$Mg${}_{x}$O${}_{3}$ films were characterised by X-ray diffraction (XRD) and X-ray reflection (XRR) using a Bruker D8 Discover with a monochromatic Cu Kα source. XRD showed only weak (012) and (110) reflexes of the CuCrO${}_{2}$ delafossite phase, indicating a poorly crystalline phase with coherent domain sizes of less than 10 nm. Cr${}_{2-x}$Mg${}_{x}$O${}_{3}$ deposited via reactive sputtering were polycrystalline, while those deposited via the ceramic pre-doped target were pseudo-amorphous. For thinner films (<100 nm), the films were homogeneous and smooth enough to measure and characterise thickness interference structures by XRR. These provided direct measurements of the film thickness and roughness consistent with ellipsometric data for the same films.

**Optical properties:** Transmission and reflection spectra were measured for all films with a PerkinElmer 650S UV-VIS spectrophotometer with integrating sphere measuring the total near normal incidence *T* and *R*. Selected well-characterised films with good electrical conductivity were also analysed by spectroscopic ellipsometry Sopra GESP 5. Ellipsometric data, transmission measurements, and the sample geometry measured by XRR were used to model the dielectric function of Cu${}_{0.4}$CrO${}_{2}$ used in all simulations above.

**Electrical measurements:** Resistance vs. temperature measurements and Hall measurements were carried out in a closed cycle refrigeration cryostat in the range of 20–300 K with magnetic field of up to 850 mT in van der Pauw geometry using gold-coated spring-loaded contacts. All voltages are probed by a Keithley 6430 sourcemeter, and Hall coefficients have been extracted from linear fits of Hall-voltage vs. magnetic field strength to minimise errors.

Seebeck measurements have been performed by clamping the sample between two copper plates as electrical contacts. One was placed on a ceramic heater, and one on a heat sink at room temperature. The Seebeck voltage upon changing the heater temperature from 300 to 320 K is plotted versus the temperature difference of the two copper terminals measured with type K thermocouples, electrically isolated but thermally connected to the copper blocks. A linear fit gives the Seebeck coefficient of the sample at 310 K.

## 5. Conclusions

Using a figure of merit ($F_{TR}$) to assess the potential of a material as p-type TCO can be helpful, but care must be taken once more simplified methods are used in the absence of reflection data. In such a case, systematic errors can affect measurements of the absorption of the material, as well as the material’s band gap. Particularly in very thin films—in the range of 20–200 nm—thickness-dependent systematic errors can lead to changes in the determined band gap and average transmission due to an overlap of thin film interference fringes with absorption structures. All these systematic measurement issues can be overcome by combining transmission with reflectance measurements. We have discussed several methods to correct the figure of merit in the absence of reflectance data by including an estimation of the normal incidence reflection and outlined simple strategies (e.g., measurements of samples with different thickness) to avoid systematic errors in the band gap determination, but also mobility and resistivity measurements.

For a robust assessment of any new material under consideration as TCO, we suggest to:

- If at all possible measure sheet resistance, transmission, *and* reflection to determine $F_{TR}$ and $E_{g}$ correctly without systematic thickness-related errors.
- If only transmission data are available:
  - estimate the refractive index using methods described in Section 2.2 and calculate $F_{TR_{0}}$
  - Measure films of different thickness to evaluate systematic errors in the band gap determination and $F_{TR_{0}}$ caused by interference fringes.
  - Measure films well above 50 nm thickness to avoid underestimating transparency due to broad reflection maximum
- Check available DFT calculations for consistency of (a) the refractive index *n*, and (b) the type of band gap. Always assess the possibility of non-direct gaps when using Tauc plots (see Section 3).
- Only give *directly measured* mobility values (DC-, AC-Hall measurements, or Seebeck measurements for polaronic materials). In all cases, follow rigorous procedures for highly resistive materials [58].
- Explicitly state the assessed films’ sample thickness and check for consistency of the thickness with interference fringes in transmission data.

Employing such strategies in screening methods will help to identify the most appropriate application for a given p-type TCO and speed up their employment in devices.

## Figures and Tables

**Table 1:** Comparison of published data for p-type transparent conducting oxides. The sample thickness, conductivity, average transmission ($T_{av}$) and, where available, average reflection were taken from the cited references. The figure of merit (FOM) $F_{TR}$ was calculated and compared to the often-used simplified version $F_{T}$. Finally, we estimated $R_{0}$ using density functional theory (DFT) values [39,41] of *n* for cases where no reflectance data were available to calculate a more representative figure of merit $F_{TR_{0}}$. The latter compares well to $F_{TR}$ in cases where measured reflectance data were available. Note: there are many other publications for the given materials; priority was given here to the most complete datasets for all relevant properties, including those in Figure 1, not on earliest publication or highest reported FOM. CSD: chemical solution deposition; MBE: molecular beam epitaxy; PLD: pulsed laser deposition.

Material	Deposition	Thickness (nm)	$T_{\mathrm{av}}$ (%)	$R_{\mathrm{av}}$ (%)	$R_{0}$ (%)	$F_{T}$ μS	$F_{{\mathrm{TR}}_{0}}$ μS	$F_{\mathrm{TR}}$ μS	Ref.
CuCrO${}_{2}$:Mg (5%)	RF sputtered	250	22.5	-	18.5	1600	2800	-	[11]
CuCrO${}_{2}$:Mg (10%)	PLD	40	40	-	18.5	350	590	-	[42]
CuCrO${}_{2}$:Mg (5%)	RF sputtered, annealed	250	35	-	18.5	25	41	-	[11]
Cu${}_{0.4}$CrO${}_{2}$	Spray pyrolysis	90	54	20	18.5	150	290	310	here
Cr${}_{2}$O${}_{3}$:Mg (8%)	MBE	180	55	15	15.5	48	82	80	[18]
Cr${}_{2}$O${}_{3}$:Mg (13%)	DC sputtered metal	192	58	16	15.5	0.4	0.6	0.6	here
a-Cr${}_{2}$O${}_{3}$:Mg (8%)	RF sputtered ceramic	50	54	35	15.5	0.15	0.25	0.8	here
LaCrO${}_{3}$:Sr (25%)	MBE	80	54	-	15.5	195	330	-	[15]
LaCrO${}_{3}$:Sr (12%)	MBE	67	63	-	15.5	52	100	-	[15]
ZnRh${}_{2}$O${}_{4}$	PLD	70	55	-	22	32	77	-	[43,44]
Cu${}_{2}$O	DC sputtered	155	26	-	17	31	50	-	[45]
SnO	e-beam evaporated	100	60	-	15.5	41	75	-	[46]
NiO	RF sputtered	150	50	-	18	14	25	-	[47]
CuAlO${}_{2}$:Mg (1%)	PLD	90	70	-	11	10	17	-	[48]
SrCu${}_{2}$O${}_{2}$:K	PLD	120	75	-	17	2.1	7.1	-	[49]
AgCrO${}_{2}$:Mg (12%)	CSD	120	60	-	21	1.9	4.6	-	[50]

## Abbreviations

The following abbreviations are used in this manuscript:

α	absorption coefficient
AC	alternating current
*c*	speed of light
CSD	chemical solution deposition
*d*	sample thickness
DFT	density functional theory
DFT-LDA	density functional theory in local density approximation
DFT-GGA	density functional theory in generalised gradient approximation
DC	direct current
$E_{1E}$	energetic position of the first interference maxima (for *R*) or minima (for *T*)
$E_{g}$	band gap
FOM	figure of merit
$F_{TR}$	FOM based on transmittance and reflectance measurements
$F_{T}$	simplified FOM based on transmittance measurements
$F_{TR_{0}}$	FOM based on transmittance measurement and reflectance estimation
GW	DFT correction based on an expansion of the single-particle
	Green’s function *G* with respect to the Coulomb interaction *W*
*h*	Plank constant
ITO	indium tin oxide
*k*	extinction coefficient
LED	light emitting device
μ	carrier mobility
MBE	molecular beam epitaxy
ν	frequency
*n*	refractive index
$n_{c}$	carrier concentration
OLED	organic light emitting diode
PLD	pulsed laser deposition
ρ	resistivity
$\rho_{d}$	density
*r*	Tauc exponent
*R*	reflectance
$R_{0}$	bulk material, normal incidence reflectance
$R_{av}$	measured, average normal incidence reflectance (1.5–3.0 eV)
RF	radio frequency
$R_{s}$	sheet resistance
σ	conductivity
SP	spray pyrolysis
$T_{av}$	measured, average normal incidence transmission (1.5–3.0 eV)
TCO	transparent conducting oxide
TFT	thin film transistor
TTFT	transparent thin film transistor
UV-VIS	ultraviolet-visible
XRD	X-ray diffraction
XRR	X-ray reflection
